# Evaluation of sodium fluoride, gluma, diode laser in dentine hypersensitivity

**DOI:** 10.6026/973206300210874

**Published:** 2025-04-30

**Authors:** Saswati Sarmah, Deepshikha Singh, Jishnu Nath, Tribeni Saikia, Anindita Bhagawati, Trishna Saikia

**Affiliations:** 1 Department of Periodontics and oral Implantology, Government Dental College, Dibrugarh, Assam, India; 2 Department of Periodontology, Rama Dental College Hospital and Research Center, Kanpur, Uttar Pradesh, India; 3 Department of Periodontology and Oral Implantology, Government Dental College, Silchar, Assam, India; 4 Department of Orthodontics and Dentofacial Orthopaedics, Government Dental College, Dibrugarh, Assam, India; 5 Department of Oral and Maxillofacial Surgery, Government Dental College, Dibrugarh, Assam, India; 6 Department of Oral Medicine and Radiology, Government Dental College, Dibrugarh, Assam, India

**Keywords:** Dentinal hypersensitivity, diode laser, visual analogue scale, gluma varnish, clinical trial

## Abstract

Dentinal hypersensitivity (DH) is one of the most painful and least predictably treated chronic conditions in dentistry. Therefore, it is of interest to evaluate the efficacy of 5% sodium fluoride (NaF) varnish, gluma varnish and diode laser and their combined application in treating dentin hypersensitivity. The findings suggest that 5% NaF alone or combined with diode laser significantly reduces the severity of DH. Combinatorial intervention of diode laser with desensitising agents is therapeutically more effective in treating DH than the application of laser, 5% NaF and Gluma alone.

## Background:

With advancements in medical facilities in this age of science and technology, people are living better lives with lower morbidity and mortality rates. Dentistry is also not an exception. Because of the development and use of novel tools and techniques, tooth loss from dental caries has decreased in modern times [1]. However, the frequency of regressive tooth alterations, such as attrition, cervical abrasion and erosion, has increased. These changes might result in dentinal hypersensitivity (DH), the most prevalent painful condition that can interfere with a person's daily life [2]. The aetiology of DH remains obscure; however, the condition is associated with exposure of the dentin from attrition or abrasion, exposed root surface due to periodontal disease or surgery and with developmental lack of a protective covering of cementum at the cementoenamel junction [3]. Sodium fluoride, strontium fluoride, formaldehyde, restorative resin, cavity varnishes and other products have been developed and recommended for treating DH [4]. Although numerous treatment modalities have been developed to treat dentin hypersensitivity, they have proven unsuccessful in the long run and/or research has produced inconsistent findings.

Laser therapy was offered as an alternative to topical desensitising medications for the treatment of DH; however, the desensitising effect appears to depend mostly on the type of laser therapy, its power and the irradiation parameters. Studies have shown that Nd: YAG, Er: YAG, CO2 and diode lasers have an effective desensitising effect; nevertheless, more investigation appears to be required to determine the ideal irradiation parameters for pulp and tubule occlusion safety [5]. Therefore, it is of interest to evaluate the efficacy of 5% sodium fluoride (NaF) varnish, gluma varnish and diode laser and their combined application in treating dentin hypersensitivity.

## Materials and Methods:

The randomized controlled clinical study was conducted for six months, including patients of both genders aged 18-40 years selected from the Department of Periodontology, Rama Dental College, Kanpur and Uttar Pradesh. One hundred eighty tooth sites were selected from 15 patients satisfying the study's inclusion criteria. Informed consent was obtained from all the study participants after detailing the procedure and advised to come for follow-up. An ethical clearance certificate was obtained from the Institutional Ethical Committee [file no- 02/IEC/RDCHRC/2017-18].

## Inclusion and exclusion criteria:

Patients complaining of severe cervical dentinal hypersensitivity, with teeth hypersensitive responding to tactile and air blast stimuli and having good health and stable mental condition were included in the study. Patients under medications, those allergic to dental products, or those with a history of gastroesophageal reflux were excluded from the study. Pregnant females and lactating mothers were also not included. Those using desensitising agents and those who had undergone periodontal surgery in the previous six months of the commencement of the study were also excluded.

## Evaluation of DH:

Hypersensitivity was tested with Airblast stimuli with a three-way syringe; the air was delivered from a standard dental unit air syringe at 40 psi (±5 psi) and 70F (±3 F). The air was directed at the exposed buccal surface of the hypersensitive tooth for two seconds from approximately 10 mm. The VAS was used to measure the pain experienced by patients in the trial and each patient's pain level was noted before the intervention was carried out. Each subject placed a vertical mark on the VAS to indicate the intensity of their level of sensitivity after the applied stimuli. After each stimulus, the degree VAS was recorded from 0 to 10 placed on a 10cm line, which corresponded to 0cm= no pain, 2 cm=mild pain, 4 cm=discomforting pain, 6 cm=distressing pain, 8 cm=horrible pain, 10 cm=excruciating pain. The VAS was evaluated at baseline, half an hour after application, at 1st month, at 3rd month and at 6 months after the intervention. All the teeth were air-dried for three seconds before treatment. The teeth sites were divided into six groups, consisting of 30 teeth each, depending on the treatment received. The groups were divided as follows.

Group 1: The 30 teeth in Group 1 were treated with a diode laser (810 nm) at a minimum distance from the tooth of 0.5cm and not more than 1.0 cm, kept perpendicular to the tooth for 1 minute and performing rapid movements apical-coronal and mesio-distal to treat the whole surface of the tooth. Each affected site received two applications of 1 minute at weekly intervals for 2 weeks.

Group 2: The teeth were treated using a colourless, aromatic fluid containing 36.1% 2- hydroxyethyl methacrylate and 5.1% glutaraldehyde (HEMA-G) in purified water (Gluma desensitiser Power Gel) applied with a micro brush for 60 seconds in the area of the lost hard dental tissue and then dried for 5 seconds with a stream of air. The procedure is repeated 2 times at weekly intervals for 2 weeks.

Group 3: The teeth were treated with 5%NaF applied with an applicator for 60 seconds, repeated 2 times at weekly intervals in direct contact on the tooth surface for 2 weeks.

Group 4: Selected teeth were treated first with Gluma desensitiser and then with diode laser following the same parameters as

Group 1: This treatment was repeated 2 times at weekly intervals for 2 weeks.

Group 5: The teeth were treated first with 5%NaF and then a diode laser was applied with the same parameters as Group 1. This application was repeated once at weekly intervals for 2 weeks.

Group 6: Group 6 is the placebo group, constituting 30 teeth selected randomly. After proper isolation, distilled water was applied using a cotton swab for 20 seconds. The procedure was repeated once at weekly intervals for 2 weeks.

## Statistical analysis:

Means and standard deviations were used to represent continuous data, whereas frequencies and percentages were used to report categorical variables. The difference in mean VAS among the treatment groups and in different follow-up periods was tested using the analysis of variance (ANOVA) test. Tuckey's honest significance test (Tuckey's HSD) was used to compare the mean difference between the two groups. The significance threshold was set as p<0.05. Statistical Package for the Social Sciences (SPSS) version 21 (IBM Corp., Armonk, NY) was used to analyse the data.

## Results:

A total of 180 teeth or sites among 15 patients were treated in the study and followed over 6 months after treatment. Significant difference was found at each interval for the control group (placebo) for all the sites analysed in the study. Among the three groups receiving a single treatment option, Group 3 (96.5%) showed the highest percentage improvement in VAS at 6 months. Among all the six groups, Group 5 (98.8%) receiving combinatorial treatment showed the highest percentage of improvement in VAS at 6 months post-treatment. The difference in mean VAS at baseline was not significantly different (p-value>0.05) among the treatment groups, implying that the values were comparable to those of the study. Among all the treatment groups, Group 5, receiving combination treatment of 5%NaF and diode laser, showed the lowest mean VAS at half an hour (0.00±0.00), at one month (0.03±0.18), at 3 months 0.03±0.18 and 6 months (0.10±0.31) post-treatment. In the case of groups receiving single treatment, Group 3, treated with 5%NaF, had the least mean VAS at half an hour (0.07±0.25), at 1 month (0.07±0.25), at 3 months (0.17±0.38) and 6 months (0.70±0.79). Notable differences (p-value<0.05) were noted in mean VAS scores at different follow-up periods among the treatment groups (Table 1). The post-hoc analysis revealed that the mean VAS value of Group 6 is significantly greater than that of all other groups (p-value<0.001) at different follow-up periods (Table 2).

## Discussion:

Although DHS is one of the most prevalent conditions faced by dental professionals, globally accepted recommendations for differential diagnosis and the selection of reliable treatment techniques are lacking [2]. The present clinical study evaluated the effectiveness of diode laser, Gluma desensitiser, 5% NaF and their combined application in treating dentin hypersensitivity. The effectiveness of lasers in treating DH was evaluated in numerous studies [6]. In the present study, the mean VAS of Group 1 treated with a diode laser significantly reduced from 8.27±0.51 at baseline to 1.53±1.20 half an hour after treatment, signifying the immediate effect of the treatment. The findings are in agreement with other similar studies [7, 8]. Potential protein coagulation in dentinal fluid could annihilate dentinal tubules, reducing hypersensitivity in the diode laser group. It could also be caused by a rise in tertiary dentine formation via enhanced odontoblastic activity [9]. However, the mean change of VAS from baseline to 6 months after laser treatment was recorded as 4.03±0.51with a percentage improvement of 48.8% only. Numerous meta-analyses and systematic reviews present conflicting evidence regarding the effectiveness of laser therapy for long-term, prolonged DH relief [10]. Gluma® Desensitizer (Heraeus Kulzer GmbH, Hanau, Germany) comprises 5% glutaraldehyde and 35% hydroxyethyl methacrylate (HEMA). Glutaraldehyde is a biological fixative that plugs dentinal tubules by nature. As HEMA is a hydrophilic monomer, it prevents pain from transmitting through fluid movements by preventing dentinal fluid proteins from coagulating inside the tubules [11]. The mean change of VAS from baseline to 6 months in Group 2 treated with Gluma varnish was 3.53±0.51, with a percentage of improvement of 42.3%. Also, we observed that the pain intensity after treatment was significantly higher in patients treated with Gluma than those treated with laser during the month's follow-up. However, at 6 months, the groups had no significant difference in mean VAS. Similar to our findings, a recent review also reported equal effectiveness of Gluma and laser in controlling DH [12]. Among the three groups receiving a single treatment option, Group 3 (96.5%), treated with 5% NaF, showed the highest percentage improvement in VAS at 6 months. The decrease in stimuli after 5%NaF application may be due to the reaction between the calcium ions of dentinal fluid and NaF, which creates calcium fluoride crystals deposited on the dentinal tubule apertures [13]. Another research has shown a significant effect of fluorides in lowering dentine hypersensitivity for up to 90 days [14]. However, in contrast to our findings, a recent clinical study reported that Gluma is more effective than NaF in reducing dentine hypersensitivity [15].

When compared with the placebo group, diode laser with the Gluma group (group 4) showed a better reduction in dentine hypersensitivity, which is statistically significant (p-value<0.001). At 1 month, 3 months and 6 months after treatment, the laser and Gluma groups showed better effectiveness than the laser alone. In contrast to our findings, another study observed that laser alone worked better in blocking open dentin tubules than laser and Gluma together [16]. Despite the melting following irradiation, it is not clinically certain that all of the dentinal tubules were occluded. This could cause certain patients to lack improvement even after multiple laser treatments. A recent study suggested that chromophore with laser improves the diode laser's ability to block dentinal tubules [17]. Among all the treatment groups, group 5, treated with 5% NaF and diode laser, showed the highest percentage (98.8%) of reduction in dentine hypersensitivity with maximum effectiveness in reducing dentinal hypersensitivity among the six groups (p-value<0.001). Numerous studies have determined that diode lasers are an effective treatment for DH, primarily when combined with NaF gel [18, 19]. Lasers assist in extending the desensitiser agent's contact with the tooth surface. Hence, combining chemical components like fluorides with lasers offers a better alternative treatment option for DH.

## Conclusion:

All the treatment options provided relief for cervical dentinal hypersensitivity-related pain except the placebo group. The 5% NaF varnish, when used alone or in combination with lasers, is significantly effective in treating cervical dentinal hypersensitivity. Among 810 nm diode laser, Gluma varnish, 5%Naf varnish and their combinations, the therapeutic effect of the combination of 5% NaF and diode laser had a very high capability to provide relief of CDH-related pain both immediately and in the long run.

## Figures and Tables

**Table 1:** Inter-group comparison of VAS score at baseline and post-treatment follow-up period data are represented as mean ± standard deviation

**VAS**	**Group 1**	**Group 2**	**Group 3**	**Group 4**	**Group 5**	**Group 6**	**p-value**
Baseline	8.27±0.58	8.37±0.56	8.37±0.61	8.47±0.51	8.53±0.51	8.63±0.49	0.122
Immediate	1.53±1.20	3.50±0.63	0.07±0.25	1.07±0.52	0.00±0.00	8.30±0.75	<0.001
1 month	3.17±0.59	4.50±0.63	0.07±0.25	1.50±0.51	0.03±0.18	8.50±0.63	<0.001
3 months	3.60±0.56	4.77±0.50	0.17±0.38	2.33±0.48	0.03±0.18	8.57±0.57	<0.001
6 months	4.90±0.88	4.83±0.46	0.70±0.79	2.40±0.50	0.10±0.31	8.57±0.57	<0.001

**Table 2:** Post-hoc analysis p-value comparison for Group-wise difference in mean VAS

	**Immediate**	**1 month**	**3 months**	**6 months**
**Groups**	**p-value for Tukey's HSD**	**p-value for Tukey's HSD**	**p-value for Tukey's HSD**	**p-value for Tukey's HSD**
Group 1 vs Group 2	<0.001	<0.001	<0.001	0.99
Group 1 vs Group 3	<0.001	<0.001	<0.001	<0.001
Group 1 vs Group 4	0.09	<0.001	<0.001	<0.001
Group 1 vs Group 5	<0.001	<0.001	<0.001	<0.001
Group 1 vs Group 6	<0.001	<0.001	<0.001	<0.001
Group 2 vs Group 3	<0.001	<0.001	<0.001	<0.001
Group 2 vs Group 4	<0.001	<0.001	<0.001	<0.001
Group 2 vs Group 5	<0.001	<0.001	<0.001	<0.001
Group 2 vs Group 6	<0.001	<0.001	<0.001	<0.001
Group 3 vs Group 4	<0.001	<0.001	<0.001	<0.001
Group 3 vs Group 5	0.99	0.99	0.85	0.003
Group 3 vs Group 6	<0.001	<0.001	<0.001	<0.001
Group 4 vs Group 5	<0.001	<0.001	<0.001	<0.001
Group 4 vs Group 6	<0.001	<0.001	<0.001	<0.001
Group 5 vs Group 6	<0.001	<0.001	<0.001	<0.001
